# Multi-walled carbon nanotubes act as a chemokine and recruit macrophages by activating the PLC/IP3/CRAC channel signaling pathway

**DOI:** 10.1038/s41598-017-00386-3

**Published:** 2017-03-22

**Authors:** Hui Li, Xiao-Qiu Tan, Li Yan, Bo Zeng, Jie Meng, Hai-Yan Xu, Ji-Min Cao

**Affiliations:** 1 Department of Physiology, Institute of Basic Medical Sciences Chinese Academy of Medical Sciences, School of Basic Medicine Peking Union Medical College, 5 Dong Dan San Tiao, Beijing, 100005 China; 2 Key Laboratory for Medical Electrophysiology, Ministry of Education, Collaborative Innovation Center for Prevention and Treatment of Cardiovascular Disease and the Institute of Cardiovascular Research, Southwest Medical University, Luzhou, 646000 China; 3 Department of Pathophysiology, Institute of Basic Medical Sciences Chinese Academy of Medical Sciences, School of Basic Medicine Peking Union Medical College, 5 Dong Dan San Tiao, Beijing, 100005 China; 4 Department of Biomedical Engineering, Institute of Basic Medical Sciences Chinese Academy of Medical Sciences, School of Basic Medicine Peking Union Medical College, 5 Dong Dan San Tiao, Beijing, 100005 China

## Abstract

The impact of nanomaterials on immune cells is gaining attention but is not well documented. Here, we report a novel stimulating effect of carboxylated multi-walled carbon nanotubes (c-MWCNTs) on the migration of macrophages and uncover the underlying mechanisms, especially the upstream signaling, using a series of techniques including transwell migration assay, patch clamp, ELISA and confocal microscopy. c-MWCNTs dramatically stimulated the migration of RAW264.7 macrophages when endocytosed, and this effect was abolished by inhibiting phospholipase C (PLC) with U-73122, antagonizing the IP3 receptor with 2-APB, and blocking calcium release-activated calcium (CRAC) channels with SK&F96365. c-MWCNTs directly activated PLC and increased the IP3 level and [Ca^2+^]_i_ level in RAW264.7 cells, promoted the translocation of the ER-resident stromal interaction molecule 1 (STIM1) towards the membranous calcium release-activated calcium channel modulator 1 (Orai1), and increased CRAC current densities in both RAW264.7 cells and HEK293 cells stably expressing the CRAC channel subunits Orai1 and STIM1. c-MWCNTs also induced dramatic spatial polarization of KCa3.1 channels in the RAW264.7 cells. We conclude that c-MWCNT is an activator of PLC and strongly recruits macrophages via the PLC/IP3/CRAC channel signaling cascade. These novel findings may provide a fundamental basis for the impact of MWCNTs on the immune system.

## Introduction

Carbon nanotubes (CNTs), a one-dimensional nanomaterial with unusual mechanical, electrical and chemical properties, have applications in many fields such as material engineering, drug delivery^1–3^, scaffolding for neuronal growth^4–7^ and bone cell proliferation^8^. The potentially extraordinary and unexpected impact of CNTs is increasingly focused on organisms or cells, including for biosecurity. Emerging studies have shown that CNTs are broad-spectrum potassium channel inhibitors^9, 10^ and inflammation inducers, and exposure to CNTs can lead to organ injuries^11–13^.

The inflammation-inducing effects of CNTs suggest that they may have an impact on immune cells. It has been found that multi-walled carbon nanotubes (MWCNTs) increase the production of H_2_O_2_ and reactive oxygen species (ROS)^14^ in RAW264.7 macrophages; inhalation of MWCNTs causes nonmonotonic systemic immunosuppression characterized by reduced T-cell-dependent antibody response and T-cell proliferative ability and decreased NK cell function^15^. Meng *et al*.^16^ reported that MWCNTs activated RAW264.7 macrophages into a M1/M2 mixed status and stimulated macrophages to secrete a large amount of MIP-1α and MIP-2 to recruit macrophages and produce angiogenesis-related cytokines. However, these findings actually depicted the downstream events of activated macrophages in response to MWCNT exposure. The upstream signaling by which MWCNTs activate the macrophages is still poorly understood, and this is the major focus of the present study.

Research has revealed that calcium mobilization is the essential event that initiates immune cell activation^17^. Increase of cytoplasmic Ca^2+^ in immune cells can lead to changes of several physiological functions, and the level and duration of cytoplasmic Ca^2+^ determines the strength and pattern of immune cell reactions to antigen stimuli. As with other cells, the calcium mobilization process in immune cells involves Ca^2+^ influx through certain Ca^2+^ channels such as the calcium release-activated calcium (CRAC) channel, and Ca^2+^ release from the endoplasmic reticulum (ER) via the Ca^2+^-releasing channel inositol 1,4,5-trisphosphate receptor (IP3R).

The CRAC channel is composed of the pore-forming calcium release-activated calcium channel modulator 1 (Orai1) subunit at the plasma membrane (PM) and the endoplasmic reticulum (ER)-resident stromal interaction molecule-1 (STIM1) auxiliary subunit, and it plays a crucial role in the calcium mobilization of immune cells^18^. ER Ca^2+^ release is mediated by the calcium-releasing channel IP3R in non-muscle cells^19^. Ca^2+^ influx and Ca^2+^ release often cross-interact. Ca^2+^ influx can induce Ca^2+^ release through the calcium-induced calcium release (CICR) mechanism, and Ca^2+^ release can also induce Ca^2+^ influx by the store-operated calcium entry (SOCE) mechanism^20^. Ca^2+^ influx through the CRAC channel is a typical example of SOCE^21^. STIM1 is a Ca^2+^ sensor of the ER. After detecting ER depletion, STIM1 translocates from the ER to the ER-PM junctions to reconstitute the CRAC channel to introduce Ca^2+^ influx^22^. We noticed in the early stage of the present study that exposure of RAW264.7 macrophages to c-MWCNTs leads to activation of CRAC channels and dramatic cell migration, and this finding inspired us to explore the action mechanisms of c-MWCNTs in recruiting macrophages.

When macrophages sense the concentration gradients of a chemokine, they start to migrate towards the chemokine by changes of cell polarity, linear motion apparatus and steering response to the outside chemokine gradients^23^. Macrophage migration is the premise of many immunological processes and can be considered a behavioral sign of macrophage activation. Now that c-MWCNTs act as a chemokine, the question is how c-MWCNTs initiate the migration process. Using several approaches including transwell assay, patch clamp recording, confocal microscopy and enzyme-linked immunosorbent assay (ELISA), we constructed a live picture illustrating the upstream signaling by which c-MWCNTs recruit macrophages, and it may suggest a fundamental basis to the impact of CNTs on macrophages and even other immune cells.

## Results

### c-MWCNTs induce dramatic migration of RAW264.7 macrophages, and this effect was abolished by inhibitors of PLC, IP3R or the CRAC channel

**Figure 1:**
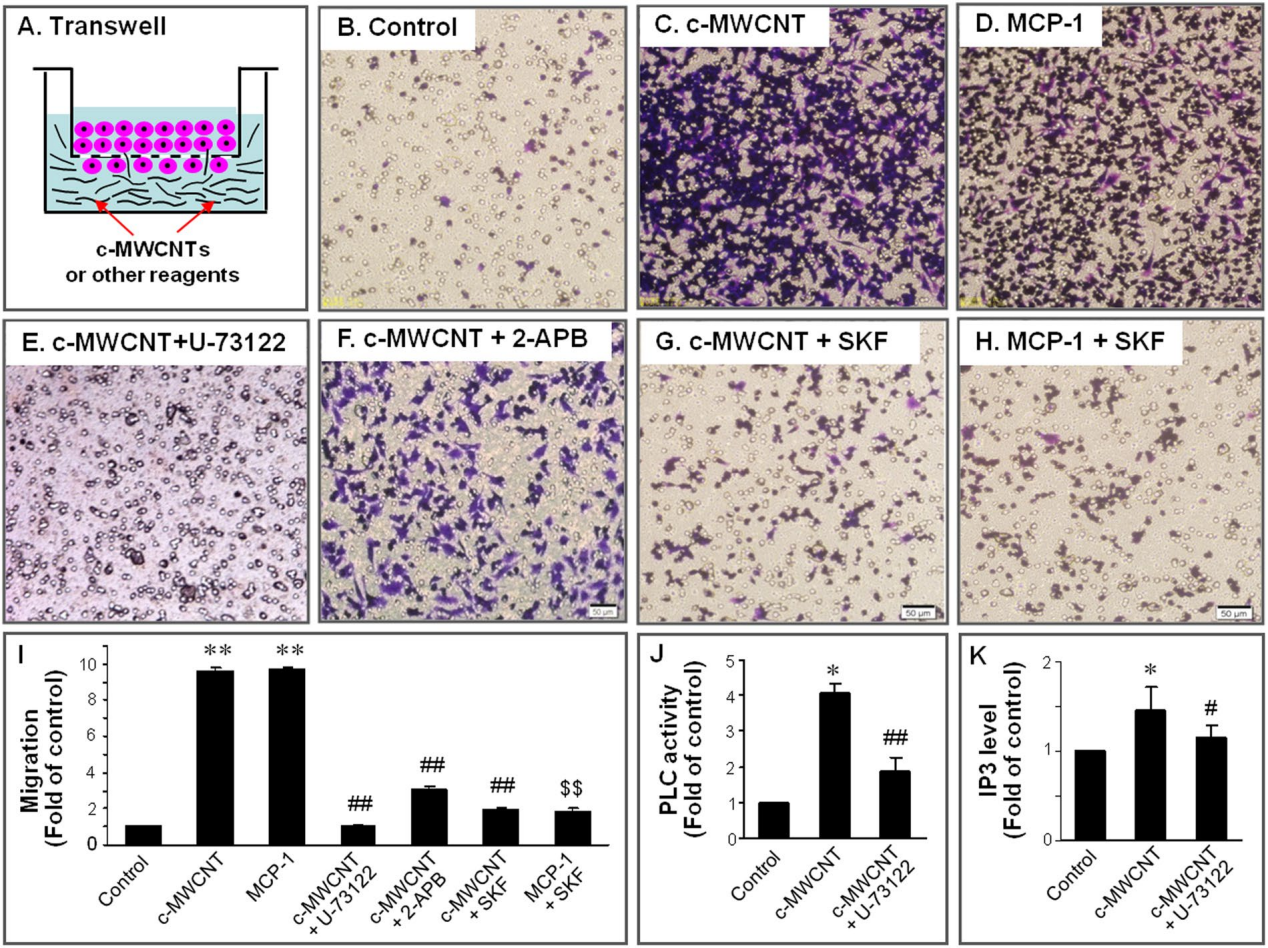
Transwell and ELISA assays showing the migrations, PLC activities and IP3 levels of RAW264.7 macrophages at different treatment conditions. (**A**) A schematic diagram of the transwell assay. (**B**) Through (**H**) crystal violet stains of migrated RAW264.7 macrophages at different treatment conditions. Migrated RAW264.7 cells were stained purple-blue. The small circles in the images were PET membrane pores that were not stained purple-blue. Note that c-MWCNTs and MCP-1 (positive control drug) strongly stimulated the migration of RAW264.7 macrophages, while pretreatment with PLC inhibitor U-73122, IP3R antagonist 2-APB, or CRAC channel blocker SK&F96365 (SKF) significantly abolished the migration-stimulating effect of c-MWCNTs. Scale bar: 50 μm. (**I**) Statistical summary of RAW264.7 cell migration at different conditions. ^**^ *P* < 0.01 *vs*. control. ^##^ *P* < 0.01 *vs*. c-MWCNT. ^$$^ *P* < 0.01 *vs*. MCP-1. N = 3 in each group. U-73122, 2-APB and SK&F96365 alone did not affect the migration (not shown). (**J** and **K**) Statistical results of ELISA assays, respectively, showing the PLC activities and IP3 levels of RAW264.7 cells at different conditions. ^**^ *P* < 0.01 *vs*. control. ^##^ *P* < 0.01 *vs*. c-MWCNT. N = 3 in each group.

In transwell studies (Fig. 1A through 1I), quiescent RAW264.7 macrophages (control) showed weak migration behavior (Fig. 1B). Carboxylated MWCNTs (c-MWCNTs) at 50 µg/ml for 24 h in the lower chamber of the transwell, which could penetrate the PET membrane pores of the upper chamber and contact the RAW264.7 cells, induced a dramatic (>9-fold) migration of RAW264.7 cells (Fig. 1C) compared with the control (Fig. 1B). Pretreatment of RAW264.7 cells with phospholipase C (PLC) inhibitor U-73122 (Fig. 1E), IP3R antagonist 2-APB (Fig. 1F), or CRAC channel blocker SK&F96365 (Fig. 1G) almost totally abolished the migration induced by c-MWCNTs. As a well-known chemokine, monocyte chemoattractant protein-1 (MCP-1) (10 ng/ml at the lower chamber) induced significant RAW264.7 cell migration (Fig. 1D), and this effect was also abolished by CRAC channel blocker SK&F96365 (Fig. 1H). A statistical summary of the RAW264.7 cell migration under different conditions is shown in Fig. 1I. U-73122, 2-APB or SK&F96365 themselves did not induce significant migration of RAW264.7 cells (not shown).

To determine whether RAW264.7 macrophages can phagocytize c-MWCNTs, we performed a transmission electron microscopy (TEM) study on the macrophages collected from the lower face of the PET membrane of the transwell insert (representing the migrated RAW264.7 cells). The TEM images (Figure S1) show that c-MWCNTs were present inside the RAW264.7 cells exposed to c-MWCNTs for 24 h, and most of them were likely wrapped by a vesicle-like structure, but a few of them directly pierced the cytoplasm. This result clearly shows that RAW264.7 macrophages can phagocytize c-MWCNTs in our experimental system.

### c-MWCNTs activate PLC and increase the IP3 level in RAW264.7 macrophages

As the macrophage-recruiting effect of c-MWCNTs can be abolished by PLC inhibitor U-73122 as shown in Fig. 1E, we speculated that PLC may be the initial molecule of the signaling chain by which c-MWCNTs stimulate macrophage migration. Consistent with this speculation, the ELISA assay showed that c-MWCNT exposure for 6 h significantly increased the PLC activities in the supernatant of RAW264.7 cell lysates in which c-MWCNTs were removed by centrifugation at 12, 000 rpm, and this effect was abolished by PLC inhibitor U-73122 in the absence of c-MWCNTs (Fig. 1J). These results strongly suggest that c-MWCNTs can directly activate PLC. Accordingly, activation of PLC resulted in an increase of the IP3 level and this effect was also inhibited by U-73122 (Fig. 1K). These results suggest that c-MWCNTs may trigger calcium mobilization by activating the PLC/IP3/IP3R signaling.

In addition, we investigated the effect of c-MWCNTs on the mRNA level with real-time quantitative PCR and found that c-MWCNT exposure for 6 h significantly increased the mRNA expression of PLC (Figure S2).

### c-MWCNTs activate CRAC channels and induce calcium mobilization in RAW264.7 macrophages

Based on the calcium mobilization hypothesis of c-MWCNTs mentioned above and the finding that blockage of CRAC channels abolished the macrophage-recruiting effect of c-MWCNTs as shown in Fig. 1G, we examined the potential effects of c-MWCNTs on the CRAC channel currents (*I*_CRAC_) using the whole-cell configuration of a patch clamp and measured the intracellular free calcium level ([Ca^2+^]_i_) using confocal microscopy in RAW264.7 macrophages.

**Figure 2:**
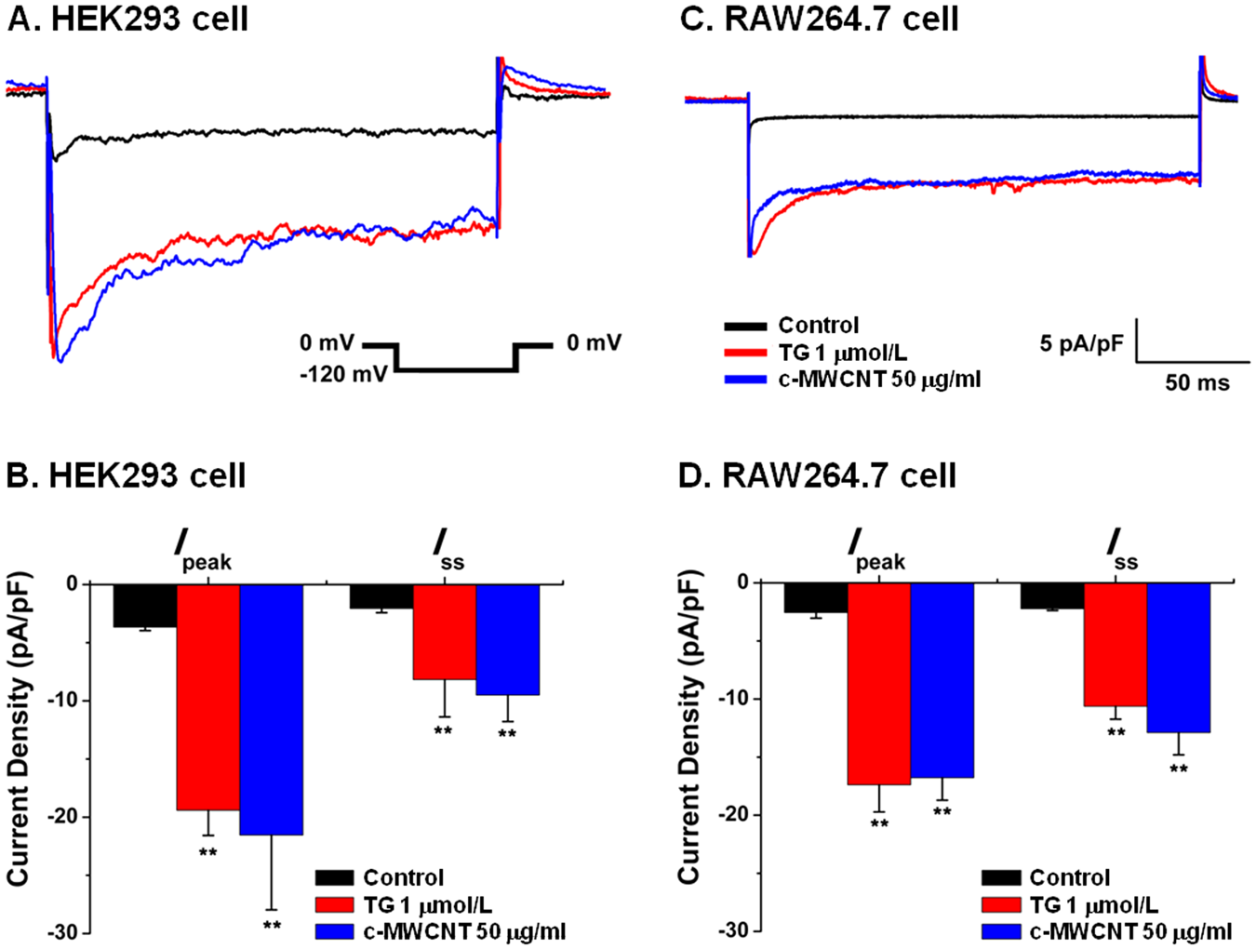
Whole-cell recordings of the CRAC currents (*I*_CRAC_) in different cells. (**A** and **B**) A representative *I*_CRAC_ and the statistical analyses of the *I*_CRAC_ densities including *I*_peak_ (peak currents) and *I*_ss_ (steady-state currents) in HEK293 cells stably expressing Orai1 and STIM1. (**C** and **D**) A representative *I*_CRAC_ and the statistical *I*_CRAC_ densities of *I*_peak_ and *I*_ss_ in RAW264.7 cells. Note that both c-MWCNTs and TG significantly increased the *I*_CRAC_ densities in the two cell lines. ^**^ *P* < 0.01 *vs*. control. N = 5 (cells) in each group. TG, thapsigargin.

The c-MWCNTs significantly increased the *I*_CRAC_ densities in HEK293 cells stably expressing Orai1 and STIM1 (Fig. 2A and B) and in RAW264.7 cells (Fig. 2C and D). We first observed that c-MWCNTs given extracellularly did not significantly affect the *I*_CRAC_ of RAW264.7 cells within 10–20 min (not shown), suggesting that c-MWCNTs act intracellularly. In HEK293 cells, c-MWCNTs (50 μg/ml) directly applied into the cytoplasm via the pipette solution soon increased *I*_CRAC_ (Fig. 2A), the peak current density (*I*_peak_) was increased from the control value of −3.66 ± 0.30 pA/pF (n = 5) to −21.54 ± 6.42 pA/pF (n = 5) (*P* < 0.05) (Fig. 2B), and the steady state current density (*I*_ss_) was increased from the control value of −2.06 ± 0.36 pA/pF (n = 5) to −9.50 ± 2.28 pA/pF (n = 5) (*P* < 0.05) (Fig. 2B). As a positive control drug, thapsigargin (TG) (1 μmol/L, exposure for 30 min) increased *I*_CRAC_, increased *I*_peak_ from the control value of −3.66 ± 0.30 pA/pF (n = 6) to −19.41 ± 2.16 pA/pF (n = 5) (*P* < 0.05), and increased *I*_ss_ from the control value of −2.06 ± 0.36 pA/pF (n = 6) to −8.17 ± 3.21 pA/pF (n = 5) (*P* < 0.05) (Fig. 2B). Similar to HEK293 cells, in RAW264.7 cells, c-MWCNTs given intracellularly through the pipette solution also quickly and significantly increased *I*_CRAC_. The peak *I*_CRAC_ densities (*I*_peak_) were increased from the control value of −2.56 ± 0.48 pA/pF to −16.77 ± 1.94 pA/pF, and the steady-state *I*_CRAC_ densities (*I*_ss_) were increased from the control value of −2.22 ± 0.16 pA/pF to −12.88 ± 1.93 pA/pF (Fig. 2C and D). TG exposure for 30 min also significantly increased the *I*_CRAC_ density in RAW264.7 cells (Fig. 2C and D).

As DMEM was used to disperse c-MWCNTs, we also observed whether the pipette solution containing 50 μl/ml DMEM (the final concentration used for dispersing c-MWCNTs in the pipette solution) without c-MWCNTs would exert an effect on *I*_CRAC_. The result showed that 50 μl/ml DMEM did not significantly affect *I*_CRAC_ compared with the pipette solution without DMEM (Figure S3)_._

**Figure 3:**
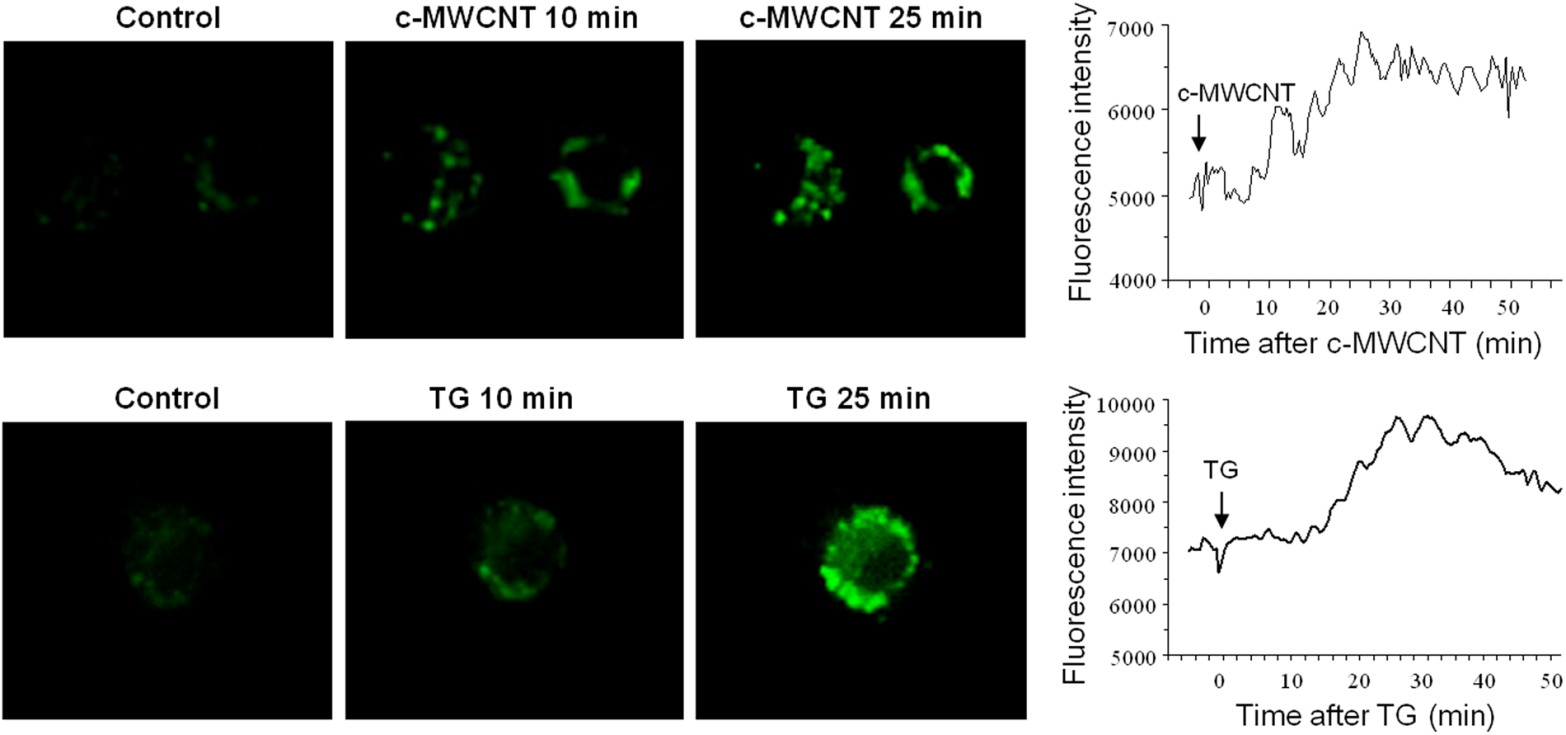
Confocal microscopy showing the calcium-mobilizing effects of c-MWCNTs (*upper row*) and the positive control drug TG (*lower row*) in RAW264.7 macrophages. Note that both c-MWCNTs and TG increased the intracellular free calcium level ([Ca^2+^]_i_) of RAW264.7 cells.

The results of confocal microscopy showed that c-MWCNTs significantly increased the [Ca^2+^]_i_ level in RAW264.7 cells (Fig. 3). As a positive control drug, TG also increased the [Ca^2+^]_i_ level in RAW264.7 cells (Fig. 3).

We also examined whether c-MWCNTs would exert an effect on the production of reactive oxygen species (ROS) in RAW264.7 macrophages. The result (Figure S4) showed that c-MWCNTs (50 μg/ml) moderately increased the ROS level. As a positive control agent in inducing ROS, H_2_O_2_ (0.5 mmol/L) strongly increased the ROS level in RAW264.7 macrophages.

### c-MWCNTs stimulate the translocation of STIM1 to the PM in HEK293 cells and RAW264.7 cells

**Figure 4:**
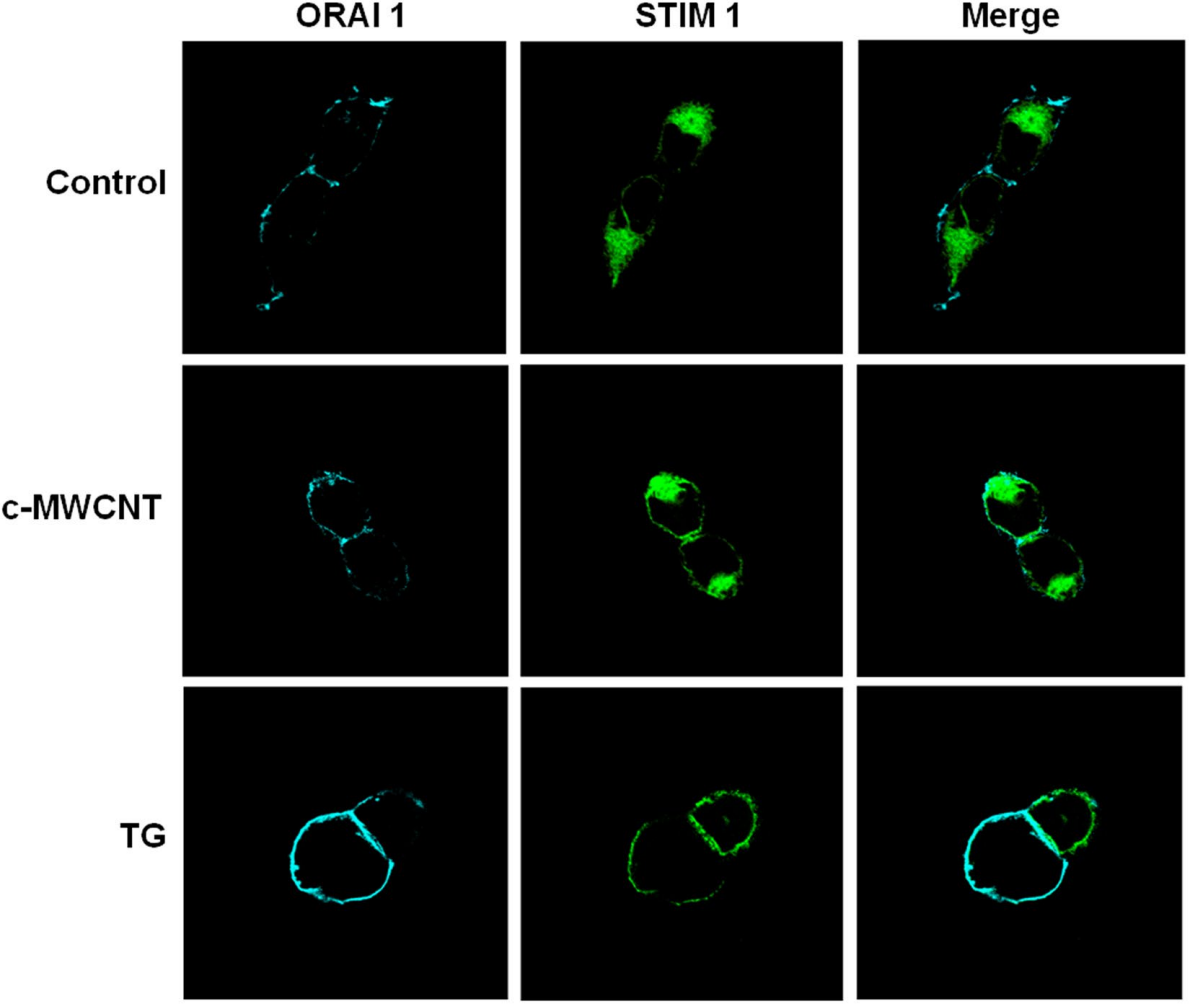
Confocal microscopy showing the cellular localization of Orai1 and STIM1 in the transfected HEK293 cells before and after treatment with c-MWCNTs (50 μg/ml, 24 h) and TG (1 μmol/L, 30 min). Note that Orai1 is uniquely located in the plasma membrane (PM). STIM1 distributed mainly in the cytoplasm and slightly in the PM at baseline (control), while it significantly translocated to the PM after treatment with c-MWCNTs or TG.

Using confocal microscopy, we demonstrated the translocation of STIM1 upon c-MWCNT exposure in HEK293 cells stably expressing EYFP-STIM1 and CFP-Orai1 and in RAW264.7 cells. In HEK293 cells, Orai1 was consistently in the PM (Fig. 4), and STIM1 was mainly distributed in the cytoplasm before exposure to c-MWCNTs (STIM1 is theoretically ER-resident at resting condition). There was a very low level of STIM1 in the PM in resting HEK293 cells, but it was diffusely distributed in the cytoplasm (Fig. 4, *upper row*). Upon c-MWCNT (50 μg/ml) stimulation for 24 h, STIM1 were obviously translocated to the PM area, as the STIM1 fluorescence signal in the PM area was significantly increased (Fig. 4, *middle row*). As a known ER-depleting agent, TG (1 μmol/L) exposure for 30 min strongly redistributed the STIM1 to the PM area (Fig. 4, *lower row*).

**Figure 5:**
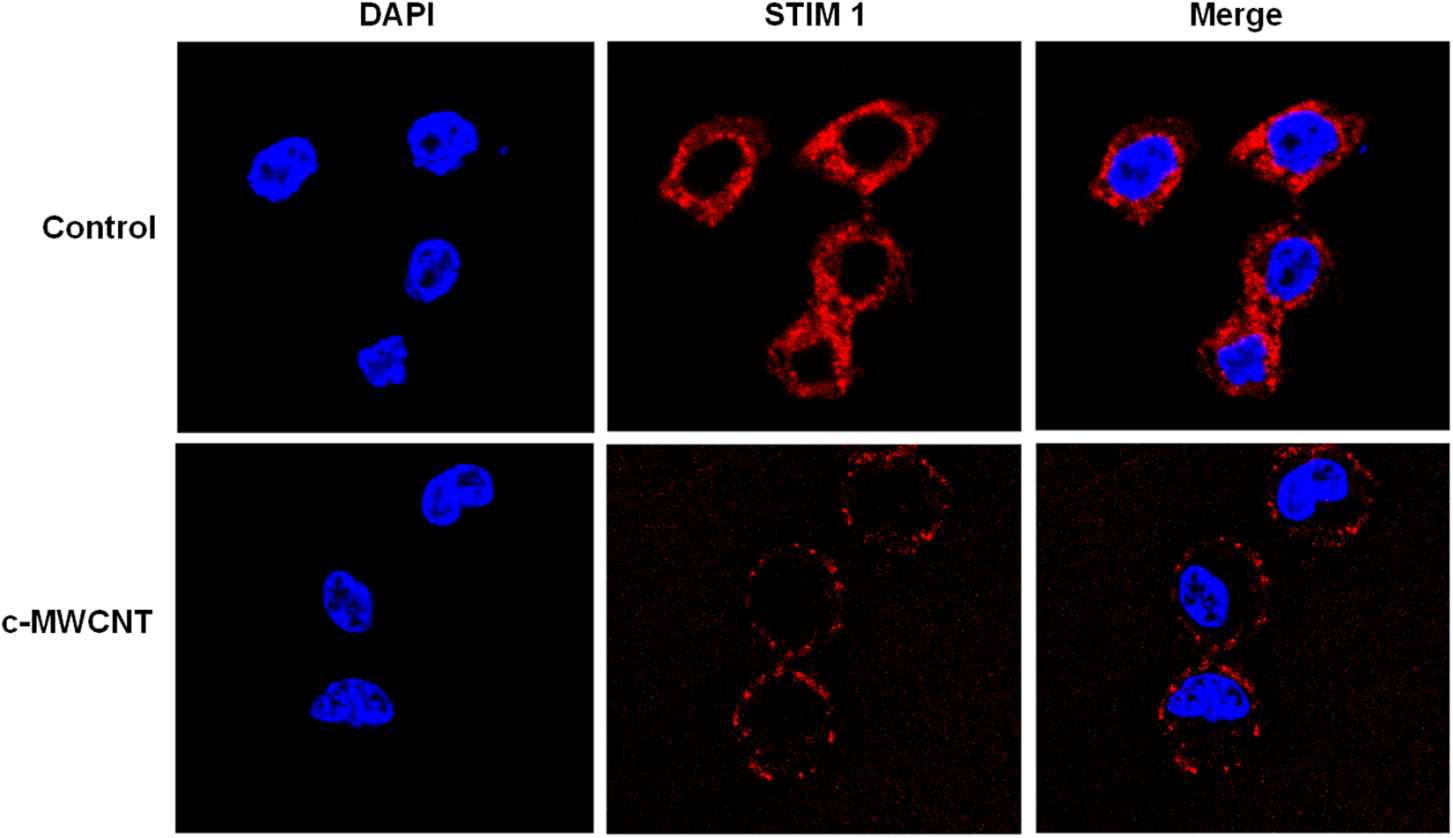
Confocal microscopy showing the translocation of STIM1 from the cytoplasm to the plasma membrane (PM) in RAW264.7 macrophages in response to c-MWCNTs (50 μg/ml, 24 h) and TG (1 μmol/L, 30 min) exposure. Both c-MWCNTs and TG strongly stimulated the translocation of STIM1 to the PM and appeared in a discrete punctate manner in the PM area.

Similar to the HEK293 cells, in resting (control) RAW264.7 cells, STIM1 was diffusely distributed in the cytoplasm (Fig. 5, *upper row*). As the ER could not be clearly recognized under a confocal microscope, it is assumed that STIM1 was diffusely distributed in the ER membrane in this condition. Exposure of RAW264.7 cells to c-MWCNTs (50 µg/ml for 24 h) led to a dramatic translocation of STIM1 to the PM area, and STIM1 distribution in the PM presented a discrete punctate manner (Fig. 5, *lower row*). The co-localization images of STIM1 and nuclei (DAPI stained) also support the translocation of STIM1 to the PM (Fig. 5). The above results suggest that c-MWCNTs stimulate the translocation of diffusely ER-resident STIM1 to the ER-PM junctions in HEK293 cells and RAW264.7 cells.

### c-MWCNTs induce spatial polarization of the KCa3.1 channel in RAW264.7 cells

**Figure 6:**
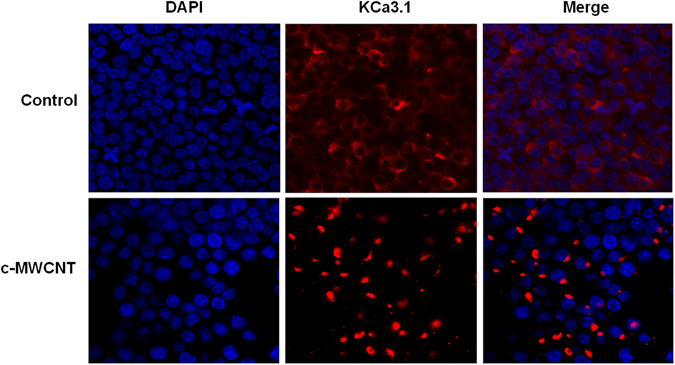
Confocal microscopic images showing the subcellular distribution of KCa3.1 channels in RAW264.7 macrophages before and after exposure to c-MWCNTs (50 μg/ml, 24 h). Note that before exposure to c-MWCNTs (control), the KCa3.1 channels were diffusely distributed, some in the PM but most in the cytoplasm of RAW264.7 cells (*upper row*). After exposure to c-MWCNTs, KCa3.1 was strikingly assembled in a polarized distribution manner in the RAW264.7 cells (*lower row*).

In the control RAW264.7 cells (before exposure to c-MWCNTs), most of the KCa3.1 channels were uniformly and diffusely distributed in the cytoplasm and some were likewise distributed in the PM (Fig. 6, *upper row*). However, after exposure to c-MWCNTs, the KCa3.1 channels were strikingly assembled to one pole of the RAW264.7 cells (Fig. 6, *lower row*). These results suggest that c-MWCNTs activate the KCa3.1 channels and induce spatial polarization of these channels, a phenomenon suggesting a role of the KCa3.1 channel in the process of polarity and steering behavior during macrophage migration.

## Discussion

**Figure 7:**
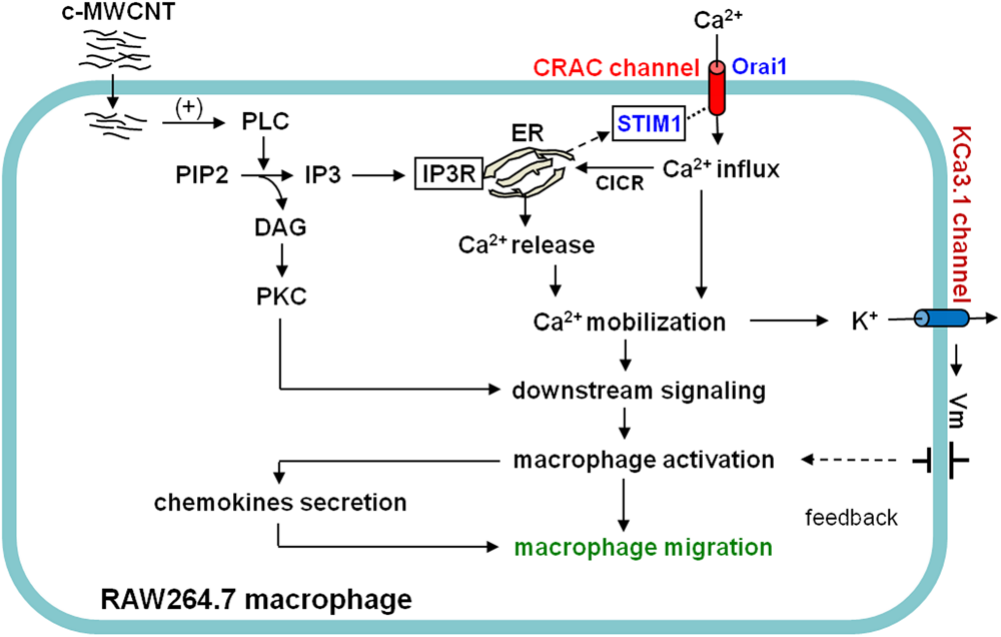
Schematic representation of the putative signaling pathways underlying the migration-stimulating effect of c-MWCNTs on RAW264.7 macrophages based on the present study and related reports. The c-MWCNTs are internalized and act intracellularly. After entering the cells, c-MWCNTs first activate PLC, leading to hydrolysis of PIP2 and production of IP3 and diacyl glycerol (DAG). IP3 triggers calcium release via the Ca^2+^-releasing channel IP3 receptor (IP3R) and leads to depletion of the endoplasmic reticulum (ER). The ER-resident STIM1 senses ER depletion and translocates to the ER-PM junctions and thus activates the Orai1 calcium channel and generates CRAC current (Ca^2+^ influx). Ca^2+^ influx, together with Ca^2+^ release, triggers the downstream signaling cascades responsible for macrophage activation and migration. Activated RAW264.7 macrophages release cytokines including chemokines, further stimulating macrophage migration. In addition, the KCa3.1 channel is activated by [Ca^2+^]_i_ elevation and mediates K^+^ efflux, thus resulting in repolarization of the membrane potential (V_m_) and providing a feedback signal to the activated macrophages.

The present study focused on the impact and mechanism of c-MWCNTs on macrophage migration. Based on the results of transwell assays, we found that c-MWCNTs act as a chemokine and strongly stimulate the migration of RAW264.7 macrophages. We further outlined the underlying upstream signaling pathways (summarized in Fig. 7), i.e., after internalization, c-MWCNTs first activated PLC, thus leading to elevation of the IP3 level in the macrophages; IP3 induced Ca^2+^ release from the ER by activating the Ca^2+^-releasing channel IP3R and thus led to ER depletion; the ER-resident STIM1 sensed the ER depletion and then translocated from the ER to the ER-PM junctions, where it reconstituted the complete CRAC channels with the membranous Orai1 and led to the CRAC channel opening and Ca^2+^ influx; Ca^2+^ release and influx resulted in Ca^2+^ mobilization and macrophage activation, then triggering the subsequent downstream signaling and driving the macrophages to undergo certain immunological activities such as migration and secretion. At the same time, the c-MWCNT-induced Ca^2+^ mobilization also activated the KCa3.1 channel and accordingly led to K^+^ efflux and a certain degree of membrane hyperpolarization, and this effect might restrict the macrophage activities to an appropriate level to avoid excessive immune responses. These findings might provide a fundamental basis for the impact of MWCNTs on macrophages and potentially even other immune cells and imply that the chemokine-like action of MWCNTs is important for further nanobiomedical practices utilizing carbon nanomaterials, both for curative and adverse effects evaluations.

Now that c-MWCNTs can recruit macrophages, we wondered what the first targeting signaling molecule for c-MWCNTs would be to initiate the migration event. By observing the reaction time course of RAW264.7 macrophages to c-MWCNT stimulation, we realized that c-MWCNTs must enter the macrophages to act. For example, in the patch clamp study, extracellular application of c-MWCNTs could not increase *I*_CRAC_ within the observed 20 min, while direct intracellular application of c-MWCNTs via the pipette solution soon increased *I*_CRAC_. We have observed similar phenomena in studying the effects of c-MWCNTs on the Kv4.2/4.3 channels in HEK293 cells and rat ventricular myocytes^10^. Extracellular application of c-MWCNTs for a longer time (for example >30 min) can increase *I*_CRAC_, suggesting that the time latency is the time needed for phagocytosis of c-MWCNTs. We also noticed in a previous study^9^ that c-MWCNTs in the PC12 cells also needed a longer time to significantly reduce the current densities of three types of K^+^ channels. These studies suggest that c-MWCNTs act at the intracellular side, and of course internalization of c-MWCNTs needs time.

The question remains: What is the first targeting signaling molecule of c-MWCNTs at the intracellular side of macrophages? We speculated and experimentally demonstrated that it is PLC, an enzyme that is the most upstream signaling molecule in controlling the Ca^2+^ mobilization in non-muscle cells, especially for explicating the action mechanism of c-MWCNTs, as c-MWCNTs are unlikely to be equipped with specific membrane receptors that react to extracellularly acting agonists. We showed that PLC inhibitor U-73122 almost totally inhibited the c-MWCNT-induced macrophage migration, a result strongly supporting the above speculation. Some other investigators have provided clues to the effects of CNTs on PLC signaling. For example, Guidetti *et al*.^24^ showed that CNTs could induce platelet activation *in vitro* through stimulation of PLC/Rap1b/integrin α(IIb)β3 signaling pathways; Matsumoto *et al*.^25^ showed that CNTs stimulated neurite outgrowth of NGF-treated neurons through the PLC/PKC/ERK signaling pathway independent of the Ras/Raf/MEK cascade. Here, we demonstrated that c-MWCNTs induced macrophage migration by targeting PLC as the most upstream signaling molecule. Meng *et al*.^16^ showed that MWCNTs stimulate the secretion of some chemokines such as macrophage inflammatory protein (MIP)-1α and MIP-2 that can recruit RAW264.7 macrophages. We thought that the chemokine secretion they observed was actually the downstream signaling in the event of MWCNT-induced migration of RAW264.7 cells. The present study mainly depicted the upstream signaling by which c-MWCNTs induced migration of RAW264.7 cells.

An additional important question emerges: Is c-MWCNT an activator of PLC or a nanozyme with PLC activity? In recent years, some nanomaterials, including carbon nanomaterials, have been found to exhibit unexpected enzyme-like activities (nanozymes) and have been considered the next-generation artificial enzymes^26, 27^. Although the abovementioned studies^24, 25^ suggest that CNTs may activate PLC, the researchers did not measure the activities of PLC of cell lysates in their studies, and thus could not answer the above question. In the present study, we showed that U-73122, a PLC inhibitor, almost totally abolished the c-MWCNT-induced migration of RAW264.7 cells (see Fig. 1E). We further identified that incubation of RAW264.7 macrophages with c-MWCNTs for 6 h directly increased the PLC activity by up to fourfold (shown in Fig. 1J) in the supernatant of cell lysates in which c-MWCNTs had been removed by centrifugation at 12, 000 rpm, and the absence of c-MWCNTs in the supernatant was confirmed by light microscopy and TEM. These studies strongly suggest that c-MWCNT is an activator of PLC, rather than an artificial nanozyme possessing the PLC activity. Overall, we identified for the first time that c-MWCNTs induce macrophage migration by activating the PLC/IP3/CRAC channel signaling cascade in RAW264.7 macrophages, and PLC is the most upstream target signaling molecule for c-MWCNTs to induce macrophage migration, and importantly, c-MWCNT is an activator of PLC. We speculate that CNTs are possibly universal PLC activators, although it still needs to be investigated whether all types of CNTs are PLC activators. This PLC-activating character of c-MWCNTs may have broad use in many industrial fields, including enzymatic degumming of plant oils^28–30^, manufacturing of biodiesels, food, nutraceuticals, detergents, leather, paper, cosmetics and even agriculture^31^. In biomedical fields, PLC and/or its manipulators may have applications in bioremediation^31^, biosensoring^32^, and microenvironmental modeling of tumors through macrophage recruitment^33, 34^. In addition, the potential for killing tumor cells using tumor-targeting MWCNTs to recruit certain type(s) of macrophages in the tumor sites merits investigation, although most recent studies on the role of macrophages in tumor development focus on the tumor-promoting effects of tumor-associated macrophages^35, 36^.

Except for the finding of the PLC/IP3/CRAC channel signaling cascade as a “forward” pathway by which c-MWCNTs induce macrophage migration, we also identified the involvement of the KCa3.1 channel, which may potentially act as a “feedback” signal in the c-MWCNT-induced behavioral event of macrophages, as the function of the KCa3.1 channel is to introduce K^+^ efflux when activated by Ca^2+^, and it thus provides a feed-forward effect on the initial influx of Ca^2+^ and preserves the negative membrane potential required for sustained Ca^2+^ influx^37^. In addition, KCa3.1 not only acts as a feedback signal for the behaviors of macrophages, but it may also facilitate the migration of macrophages cooperating with other channels such as Kv1.3 and CRAC channels. The dramatic cellular polarization of KCa3.1 in RAW264.7 macrophages that we observed in response to c-MWCNT exposure suggests the activation of the KCa3.1 channel and its certain roles in the migration process of macrophages, potentially including the formation of protruding and cell polarity. KCa3.1 has been found to promote the migration of other types of cells, including human lung mast cells^38^, human fibrocytes^39^ and mouse neuroblasts *in vivo* ^40^. The present study further identified the involvement of KCa3.1 in the c-MWCNT-induced migration of RAW264.7 macrophages. However, the exact role of KCa3.1 in c-MWCNT-induced macrophage migration needs further investigation.

Although we demonstrated the key upstream signaling by which c-MWCNTs triggered the macrophage migration, some other effects of c-MWCNTs on the macrophages, such as the enhancement of PLC gene transcription and ROS production that we showed, might be involved in the migration event or other activities of macrophages.

In summary, the present study demonstrated that c-MWCNTs act as a chemokine and strongly stimulate the migration of RAW264.7 macrophages by activating the PLC/IP3/CRAC signaling pathway. In particular, PLC serves as the most upstream targeting signaling molecule of c-MWCNTs, and c-MWCNT is an activator of PLC. The KCa3.1 channel is also involved in this event. These findings may shed light on the biomedical impact of CNTs at both the theoretical and practical level.

## Methods

### Reagents

Reagents were purchased from the following suppliers: monocyte chemotactic protein-1 (MCP-1), SK&F96365, 2-aminoethyl diphenylborinate (2-APB), NP-40, Cs aspartate, CsOH, EGTA, HEPES, anti-beta actin antibody, 4′,6-diamidino-2-phenylindole (DAPI), thapsigargin (TG) and mouse anti-KCa3.1 antibody (Sigma, USA); ryanodine and DyLight 650-conjugated donkey anti-mouse secondary antibody (Abcam, UK); rabbit anti-Orai1 antibody (Alomone Labs, Israel); mouse anti-STIM1 antibody (Abnova, USA); bovine serum albumin (BSA) (Bovogen, Australia); anti-fluorescence quenching agent (Dako, Denmark).

### Preparation and characterization of MWCNTs

We used the carboxylated MWCNTs (c-MWCNTs) in this study, as this type of CNT has a good hydrophilic property and can disperse well in the aqueous phase. The c-MWCNTs were developed from the pristine MWCNTs (p-MWCNTs). The p-MWCNTs were purchased from Chengdu Organic Chemicals Co. Ltd. (Chengdu, China) and the c-MWCNTs were prepared as previously described^41^. In brief, the c-MWCNTs were synthesized based on the p-MWCNTs through a combined oxidation procedure and probe sonication. The as-received MWCNTs were dried at 50 °C in a vacuum oven overnight and then were suspended in a 3:1 (volume) mixture of concentrated H_2_SO_4_/HNO_3_ and sonicated with a power of 750 W for 80 sec. The resulting mixture was diluted with a large amount of distilled water, filtered and rinsed thoroughly until the pH was neutral before drying to constant weight at 50 °C in a vacuum oven. The prepared c-MWCNTs were subjected to scanning electron microscopy (SEM, Hitachi S-5200) to characterize their morphology. The length distribution for the c-MWCNTs was obtained by counting more than 300 nanotubes randomly taken in ten SEM images. X-ray photon spectroscopy (XPS, Japan JEOL Scientific JPS-9010TR) and Fourier transform infrared spectroscopy (FTIR, Necolet NEXUS 670) were applied to analyze their surface chemistry. The powder sample of c-MWCNTs was sterilized by autoclaving and then dispersed in Dulbecco’s modified Eagle’s medium (DMEM) to obtain a stock solution of 1 mg/ml by the aid of probe sonication (sonication time: 60 sec; working mode: working 3 s following 3 s stop; working power: 600 watt). For cell studies, c-MWCNTs were diluted in DMEM at a concentration of 50 μg/ml. The physicochemical characteristics of c-MWCNTs were previously described^42^. The average length of pristine MWCNTs was 50 μm. The length distribution for c-MWCNTs ranged from 300 nm to 1.5 μm, and the average length was 926–945 nm depending on the manufacturing batch. The FTIR spectrum of c-MWCNTs showed that the characteristic absorption of the carboxylic group at 1720 cm^−1^ is substantially decreased in the c-MWCNT spectrum, while an absorption peak at 1630 cm^−1^ appeared.

### Evaluation of potential endotoxin contamination in the MWCNTs

To inactivate the potentially contaminated endotoxin in the c-MWCNT samples, the above prepared c-MWCNTs were routinely autoclaved (temperature >250 °C) and were again heated in an oven at 250 °C for 45 min prior to the experiment, with the suggested procedures of the International Pharmacopoeia (Sixth Edition, 2016) as a reference. The potential presence of endotoxin in the c-MWCNT solution was further evaluated using the limulus amebocyte lysate (LAL) gel clot assay kit (sensitivity 0.125 EU/ml) (Houshiji Company, Xiamen, China). To perform the assay, c-MWCNTs were added into the endotoxin-free water, vortexed and shaken for 15 min, followed by centrifugation at 12,000× *g* for 10 min. The supernatant was collected and added to the LAL assay solution (v/v = 1:1). The mixture was incubated at 37 °C for 1 h. Then, the tube was inverted to observe gel clot formation. Negative results were obtained with the c-MWCNT solution, demonstrating that the c-MWCNTs we used were endotoxin-free. The endotoxin of 0.25 EU/ml was set as a positive control which could induce gel clot formation. Because c-MWCNTs produced a black solution, we did not use a colorimetric test to examine endotoxin contamination in the c-MWCNT solution, out of concern that it may yield a false-positive result.

### Cell culture and transfection

The RAW264.7 macrophage-like murine cell line (ATCC Number TIB-71; American Type Culture Collection, US) was purchased from the Cell Center, Peking Union Medical College. Cells were cultured at 37 °C and 5% CO_2_ humidified atmosphere in Dulbecco’s modified Eagle’s medium (DMEM) supplemented with 10% fetal bovine serum (FBS), 1 unit/ml penicillin, 100 mg/ml streptomycin. Cultured RAW264.7 cells were used for the transwell assay, confocal microscopy and patch clamp study.

Plasmids encoding mCFP-Orai1 and STIM1-EYFP were constructed according to the method described by Zeng *et al*.^43^. T-REx HEK293 cells (Invitrogen) were routinely cultured and transfected with the above two plasmids to obtain stable expression of Orai1 and STIM1 in this cell line. Transfected HEK293 cells were grown in a DMEM complete culture medium with 200 ng/ml zeocin and 1 μg/ml tetracycline to induce Orai1 expression.

### Transwell assay

Transwell assays were performed to evaluate the effect of c-MWCNTs on the migration capacity of RAW264.7 cells. The transwell apparatus (Millipore Co., USA) is composed of an upper chamber (Millipore insert) and a lower chamber. The bottom of the upper chamber is a polyethylene terephthalate (PET) membrane with 8-μm pores that allow cells to penetrate/migrate. The lower chamber is a well of a 24-well culture plate. Cultured RAW264.7 cells were collected and suspended in DMEM medium, and then were counted and adjusted to a concentration of approximately 10^5^ cells/ml DMEM. Cell suspensions (200 μl) were seeded onto the upper chamber and cultured in DMED with 10% FBS at 37 °C for 24 h to allow cells to fully grow and adhere to the upper face of the PET membrane so that they would be ready to migrate. The lower chamber was filled with 700 μl DMEM. c-MWCNTs or other agents (such as MCP-1, U-73122, etc.) were delivered into the DMEM solution contained in the lower chamber. Cells that migrated across the pores of the PET membrane would adhere to the lower face of the PET membrane of the Millipore insert. Note that although c-MWCNTs were placed in the lower chamber, the 8-μm pores of the PET membrane equipped in the bottom of the Millipore insert also allowed c-MWCNTs to penetrate the pores and diffuse from the lower chamber to the upper chamber driven by the c-MWCNT gradient between the two chambers, therefore providing a chance for RAW264.7 cells in the upper chamber to be exposed to c-MWCNTs and thus trigger cell migration. After the 24-h migration, the Millipore insert was removed and the DMEM medium was discarded. The insert (including the PET membrane) was washed with PBS three times, and cells adhering to the lower face of the PET membrane (indicating the migrated cells) were fixed with 4% paraformaldehyde for 15 min, and then were stained with 0.1% crystal violet for 20 min, followed by a PBS wash to remove excess stain. Cells on the upper face of the PET membrane (cells not migrated) were removed by a gentle wipe with a cotton swab. Cells adhering to the lower face of PET membrane (cells migrated) were counted under a microscope (200× objective). Five fields were randomly chosen and cells were captured and counted with Image-pro Plus 6.0 with the amount of normal control cells standardized to 1. The same experiments were performed in triplicate in three wells.

## Transmission electron microscopy (TEM)

TEM was performed to evaluate the phagocytosis of RAW264.7 macrophages on c-MWCNTs according to our recent report^44^ with modifications. Briefly, after the transwell experiments, RAW264.7 cells were collected from the lower face of the PET membrane of the insert (representing the migrated cells). The collected RAW264.7 cells in DMEM were centrifuged at 1000 rpm for 4 min. The cell pellets were fixed with 2.5% glutaraldehyde in 0.1 mol/L phosphate buffer (pH 7.2) and stored at 4 °C until TEM was performed. To perform the TEM, RAW264.7 cells were washed with phosphate buffer and were further fixed with 1% phosphate buffered osmium tetroxide, rewashed with phosphate buffer, dehydrated by graded ethanol and acetone, infiltrated with epoxy resin and acetone, and finally were embedded in epoxy resin. Ultra-thin sections were cut with an RMC MT-X ultrathin microtome, mounted on copper grids and then stained with uranyl acetate and lead citrate and washed with distilled water. Ultrastructural images of RAW264.7 cells were shot with a GATAN digital camera (Gatan, USA) equipped in a transmission electron microscope (JEOL-1011, Japan). RAW264.7 cells without exposure to c-MWCNTs served as the control.

### Patch clamp

Whole-cell patch clamp experiments were performed to record the CRAC currents (*I*_CRAC_) of cultured RAW264.7 cells and HEK293 cells with stable expression of CRAC channels (Oria1 and STIM1 subunits) at room temperature using an EPC-10 amplifier (HEKA, Germany). Pipette resistance was controlled to 2–4 MΩ. To record *I*_CRAC_, a 200-ms step pulse was delivered from a holding potential of 0 mV to −120 mV. The bath (external) solution consisted of (in mmol/L): NaCl 155, KCl 4.5, CaCl_2_ 22, MgCl_2_ 1, D-glucose 10, HEPES 5 (pH 7.4 with NaOH). The pipette (internal) solution consisted of (in mmol/L): Cs aspartate 140, MgCl_2_ 3.01, CaCl_2_ 0.66, EGTA 11.68 (free [Ca^2+^]_i_ was approximately 10 nM), HEPES 10 (pH 7.2 with CsOH). Current data were analyzed using Clampfit 10.1 software, and current tracing generation was performed by OriginPro8.0 software. The c-MWCNTs were added to the pipette solution to obtain the desired concentration (50 μg/ml), thus yielding an intracellular application of c-MWCNTs to observe the “immediate” effect of c-MWCNTs on *I*_CRAC_, as the effect of extracellular c-MWCNTs on *I*_CRAC_ emerged very slowly (>30 min).

### Confocal microscopy

Confocal microscopy was used to visualize the subcellular localization of Orai1 and STIM1 both in RAW264.7 cells and HEK293 cells stably expressing Orai1 and STIM1, and KCa3.1 in RAW264.7 cells. After incubation with c-MWCNTs (dispersed in DMEM) for 24 h, both types of cells were fixed with 4% paraformaldehyde, washed with PBS, blocked with BSA, and then incubated with antibodies, respectively, against Orai1 (1:500), and STIM1 (1:500) or KCa3.1 (1:500) at 4 °C overnight. Cells were then washed thrice with PBS, followed by incubation with DyLight650-conjugated donkey anti-mouse secondary antibody for 1 h. Cells were finally washed with PBS, stained with DAPI (10 μg/ml) for 10 min, washed with PBS, and cover sealed with anti-fluorescence quenching agent. Visualization and a photo shot of the fluorescence for Orai1, STIM1 and KCa3.1 were performed under a confocal microscope (Olympus, Japan). The excitation/emission wavelengths (nm) for DAPI, CFP, EYFP and DyLight650 were, respectively, 358/461, 433/476, 513/527 and 654/673.

Confocal microscopy was also used to evaluate the calcium mobilizing effect of c-MWCNTs in RAW264.7 cells. Briefly, RAW264.7 cells were incubated with Fluo-3/AM (3 μmol/L) in the dark for 1 h, washed with PBS three times, and then placed into DMEM containing c-MWCNTs. Cell fluorescence change was continuously monitored under a confocal microscope until the Ca^2+^ transient appeared.

### Enzyme linked immunosorbent assay (ELISA)

ELISA was performed to measure the activity of PLC and the level of IP3 in RAW264.7 macrophages in response to c-MWCNT exposure, using the respective ELISA kits (TSZ Biosci., USA) for PLC and IP3. Briefly, after exposure to c-MWCNTs (given extracellularly) for 6 h, RAW264.7 cells were washed and then were lysed. Cell lysates were collected and centrifuged at 12,000 rpm to acquire the supernatant free of MWCNTs. The absence of c-MWCNTs in the supernatant was confirmed by light microscopy and TEM. ELISA was performed to measure the activity of PLC and the level of IP3 in the cell lysate supernatant according to the manufacturer’s instructions. Optical absorbing values were read at 450 nm and corrected by 570 nm. The data were analyzed using SoftMax Pro4.8 software.

### Real time quantitative PCR (RT-qPCR)

RT-qPCR was performed to evaluate the potential effect of c-MWCNTs on the transcription level of PLC in RAW264.7 cells. Briefly, RAW264.7 cells were cultured in DMEM containing c-MWCNTs in a 6-cm dish and harvested at a cell density of 80–90%. Total RNA was isolated from the RAW264.7 cells using an RNA extraction kit (Tiangen Co., Beijing, China). The first strand cDNA was created with 1 μg total RNA using ReverTra Ace qPCR RT Kit (Toyobo, Japan). Primers were designed using the Premier 5.0 program. PLC, sense: ATGAAATCCTTTACCCACC, anti-sense: ACAGCGACATCCAGACA. β-actin, sense: TTGTTACCAACTGGGACGACAT, anti-sense: GTGTTGAAGGTCTCAAACATGATCT. RT-qPCR was performed using Real-time PCR Master Mix (Toyobo, Japan) with the ABI-7900 Real Time PCR System (ABI, USA). Each measurement was made in triplicate and expressed relative to the housekeeping gene β-actin.

### Measurement of ROS level

To determine whether c-MWCNTs induce production of reactive oxygen species (ROS) in RAW264.7 cells, intracellular ROS was measured using an ROS assay kit (Jiancheng Biotechnology, Nanjing, China), based on the ROS-mediated conversion of nonfluorescent 2,7-dichlorofuorescin diacetate (DCFH) into fluorescent dichlorofluorescein (DCF). After incubation with different treatments in 96-well plates, RAW264.7 cells were washed with PBS and subsequently incubated with DCFH-DA (final concentration 10 μmol/L) in PBS at 37 °C for 20 min. Then, the fluorescence of the cells was measured using flow cytometry (BD, USA).

### Statistical analysis

The data are presented as the mean ± standard deviation (SD). Statistical analyses were performed using Student’s *t*-test for paired data and analysis of variance followed by Newman-Keuls multiple comparison for multiple group data. *P* < 0.05 was considered statistically significant.

## Electronic supplementary material

Supplementary figures and legends
