# Supplementary material for: Multi-walled carbon nanotubes act as a chemokine and recruit macrophages by activating the PLC/IP3/CRAC channel signaling pathway
Source: Sci Rep. 2017 Mar 22;7:226. doi: 10.1038/s41598-017-00386-3 (PMC5428205; doi:10.1038/s41598-017-00386-3)
Supplement: Supplementary file 1 — Supplementary figures and legends [file 41598_2017_386_MOESM1_ESM.doc]

**Supplementary information**

**Multi-walled carbon nanotubes act as a chemokine and recruit macrophages by activating the PLC/IP3/CRAC channel signaling pathway**

Hui Li^1,2^, Xiao-Qiu Tan^2^, Li Yan^3^, Bo Zeng^2^, Jie Meng^4^, Hai-Yan Xu^4^, Ji-Min Cao^1^

^1^ Department of Physiology, Institute of Basic Medical Sciences Chinese Academy of Medical Sciences, School of Basic Medicine Peking Union Medical College, 5 Dong Dan San Tiao, Beijing 100005, China.

^2^ Key Laboratory for Medical Electrophysiology, Ministry of Education, Collaborative Innovation Center for Prevention and Treatment of Cardiovascular Disease and the Institute of Cardiovascular Research, Southwest Medical University, Luzhou 646000, China.

^3^ Department of Pathophysiology, Institute of Basic Medical Sciences Chinese Academy of Medical Sciences, School of Basic Medicine Peking Union Medical College, 5 Dong Dan San Tiao, Beijing 100005, China.

^4^ Department of Biomedical Engineering, Institute of Basic Medical Sciences Chinese Academy of Medical Sciences, School of Basic Medicine Peking Union Medical College, 5 Dong Dan San Tiao, Beijing 100005, China.

* Corresponding author (email: [caojimin@126.com](mailto:caojimin@126.com))

**Supplementary figures and legends**


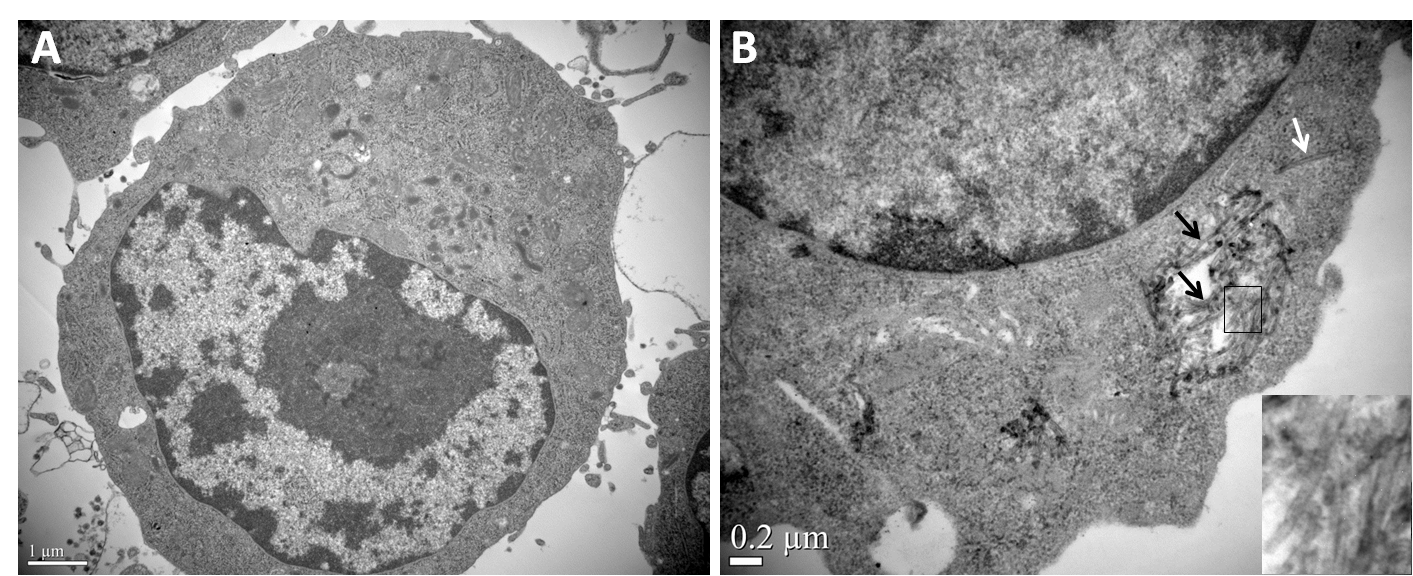


Figure S1. TEM images showing the phagocytosis of carboxylated MWCNTs (c-MWCNTs) by RAW264.7 macrophages. A, RAW264.7 cells without exposure to c-MWCNTs, no c-MWCNT was found inside the cell. B, RAW264.7 cells exposed to c-MWCNTs for 24h, c-MWCNTs were found phagocytized by the RAW264.7 cell. Most of the c-MWCNTs were wrapped in a vesicle-like structure (suggesting phagocytosis) (black arrows), but a few of c-MWCNTs directly pierced the cytoplasm (white arrow).


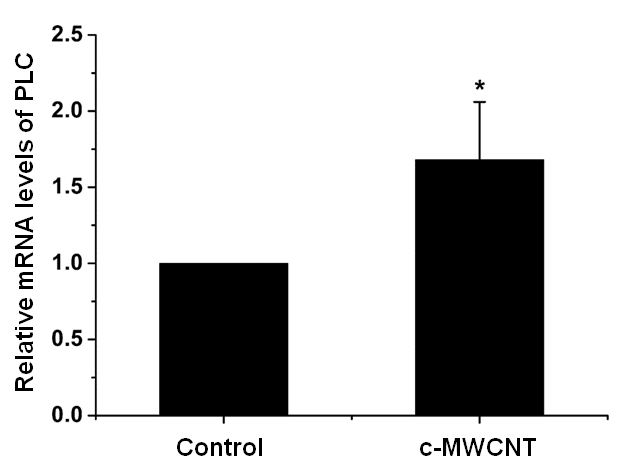


Figure S2. RT-qPCR assay showing the effect of c-MWCNTs on the mRNA level of PLC in RAW264.7 macrophages. Note that c-MWCNTs increased the PLC mRNA level. β-actin was used as the house-keeping gene. * *P* < 0.05 *vs.* control. N = 6.


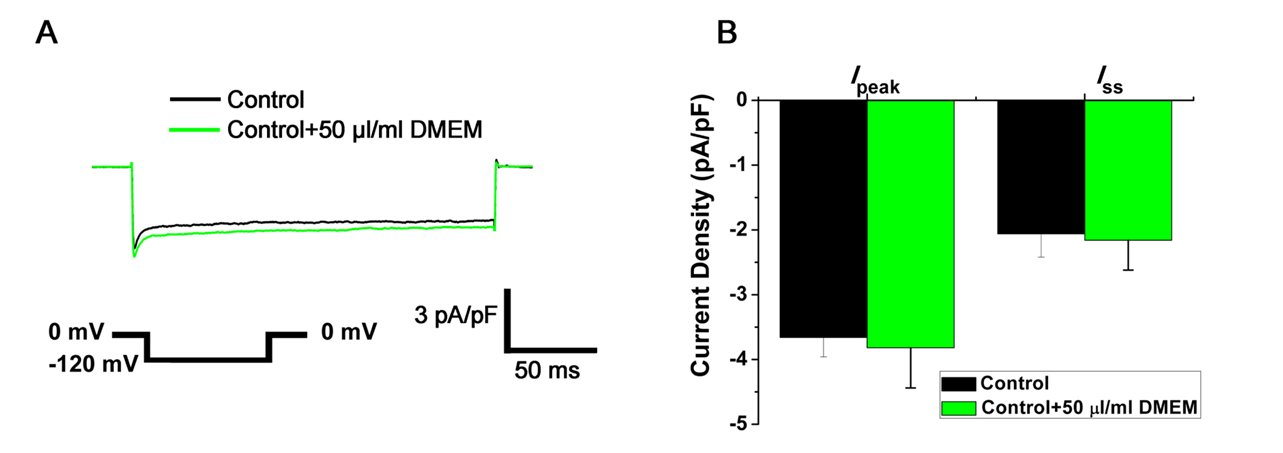


Figure S3. The effect of DMEM in the pipette solution on the *I*_CRAC_ in HEK293 cells steadily expressing Orai1 and STIM1. A, the original current traces of *I*_CRAC_ elicited by a 200-ms step pulse from the 0-mV holding potential to -120 mV. Control, normal pipette solution. Control + 50 μl/ml DMEM, pipette solution containing DMEM (but without c-MWCNTs). DMEM of 50 μl/ml was the final concentration of DMEM for dispersing c-MWCNTs in the pipette solution. The result showed that DMEM had no effect the *I*_CRAC_ compared with the normal pipette solution. N = 4 cells in each group.


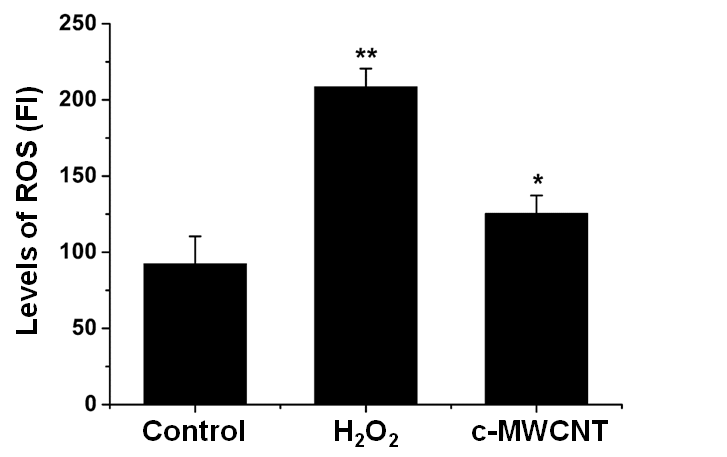


Figure S4. The effect of c-MWCNTs on the intracellular ROS level in RAW264.7 macrophages. c-MWCNTs (final concentration 50 μg/ml) moderately increased the ROS level compared with the control (DMEM culture medium). As a positive control, H_2_O_2_ (0.5 mmol/L) strongly increased the ROS level. * *P* < 0.05, ** *P* < 0.01 *vs.* control. N = 10 in each group. FI, fluorescence intensity.
